# RIG-I agonist SLR10 promotes macrophage M1 polarization during influenza virus infection

**DOI:** 10.3389/fimmu.2023.1177624

**Published:** 2023-07-05

**Authors:** Wenxin Wu, Wei Zhang, Jeremy S. Alexandar, J. Leland Booth, Craig A. Miller, Chao Xu, Jordan P. Metcalf

**Affiliations:** ^1^Pulmonary, Critical Care and Sleep Medicine, Department of Medicine, University of Oklahoma Health Sciences Center, Oklahoma City, OK, United States; ^2^Department of Veterinary Pathobiology, College of Veterinary Medicine, Oklahoma State University, Oklahoma State University, Stillwater, OK, United States; ^3^Department of Biostatistics and Epidemiology, University of Oklahoma Health Sciences Center, Oklahoma City, OK, United States; ^4^Pulmonary Section, Medicine Service, Veterans Affairs Medical Center, Oklahoma City, OK, United States; ^5^Department of Microbiology and Immunology, University of Oklahoma Health Sciences Center, Oklahoma City, OK, United States

**Keywords:** influenza virus, RIG-I, agonist, SLR10, smoking, innate immunity, macrophage polarization

## Abstract

**Rationale:**

A family of short synthetic, triphosphorylated stem-loop RNAs (SLRs) have been designed to activate the retinoic-acid-inducible gene I (RIG-I) pathway and induce a potent interferon (IFN) response, which may have therapeutic potential. We investigated immune response modulation by SLR10. We addressed whether RIG-I pathway activation with SLR10 leads to protection of nonsmoking (NS) and cigarette smoke (CS)-exposed mice after influenza A virus (IAV) infection.

**Methods:**

Mice were given 25 µg of SLR10 1 day before IAV infection. We compared the survival rates and host immune responses of NS and CS-exposed mice following challenge with IAV.

**Results:**

SLR10 significantly decreased weight loss and increased survival rates in both NS and CS-exposed mice during IAV infection. SLR10 administration repaired the impaired proinflammatory response in CS-exposed mice without causing more lung injury in NS mice as assessed by physiologic measurements. Although histopathologic study revealed that SLR10 administration was likely to result in higher pathological scores than untreated groups in both NS and CS mice, this change was not enough to increase lung injury evaluated by lung-to-body weight ratio. Both qRT-PCR on lung tissues and multiplex immunoassay on bronchoalveolar lavage fluids (BALFs) showed that most IFNs and proinflammatory cytokines were expressed at lower levels in SLR10-treated NS mice than control-treaded NS mice at day 5 post infection (p.i.). Remarkably, proinflammatory cytokines IL-6, IL-12, and GM-CSF were increased in CS-exposed mice by SLR10 at day 5 p.i. Significantly, SLR10 elevated the ratio of the two chemokines (CXCL9 and CCL17) in BALFs, suggesting macrophages were polarized to classically activated (M1) status. *In vitro* testing also found that SLR10 not only stimulated human alveolar macrophage polarization to an M1 phenotype, but also reversed cigarette smoke extract (CSE)-induced M2 to M1 polarization.

**Conclusions:**

Our data show that SLR10 administration in mice is protective for both NS and CS-exposed IAV-infected mice. Mechanistically, SLR10 treatment promoted M1 macrophage polarization in the lung during influenza infection. The protective effects by SLR10 may be a promising intervention for therapy for infections with viruses, particularly those with CS-enhanced susceptibility to adverse outcomes.

## Introduction

The innate immune system provides immediate protection against infection by recognizing and responding to pathogens in a non-specific manner. The innate responses to influenza A virus (IAV) are initiated by recognition of pathogen-associated molecular patterns (PAMPs) by host’s pattern recognition receptors (PRRs), such as retinoic acid-inducible protein I (RIG-I) (1) and Toll-like receptors (TLRs). Cigarette smoke (CS) increases the risk of influenza hospitalization and reduces flu vaccine efficiency in older people (2), yet the mechanisms are not fully understood. CS suppresses host antiviral defense in the immune system (3–5). We have shown that CS exposure decreased the survival rates in wild type (WT) mice infected with IAV. The increased mortality was associated with a suppressed innate immune response in the lungs (6).

Macrophages are essential components of innate immunity and are commonly involved in viral infections and antiviral states. In the case of IAV, animal studies showed that macrophage depletion results in increased viral replication, with higher lung inflammation responses and increased mortality (7–9). During pathogenic infection, macrophages demonstrated plasticity *via* activation into two polarized phenotypes, classically activated (M1) and alternatively activated (M2) macrophages (10). The M1 macrophages are mainly polarized by T helper type 1 (Th1) cytokines and pro-inflammatory cytokines, such as granulocyte-macrophage colony-stimulating factor (GM-CSF), IL-6, IL-1β, and IL-12. In comparison, M2 macrophages are polarized by IL-13, IL-4, macrophage colony-stimulating factor (M-CSF), and glucocorticoids and serotonin (10). Functionally, M1 macrophages exhibit microbicidal activity and M2 macrophages facilitate clearance of parasites, enhance tissue repair, and promote wound healing. Once polarized, M1 or M2 macrophages secrete a series of cytokines to stimulate or suppress inflammatory responses (11). M1 macrophages have an elevated ability to release proinflammatory cytokines, such as IL-1β, IL-6, and IL-12, and chemokines such as interferon gamma-induced protein 10 (IP-10) and CXCL9. Conversely, M2 macrophages release anti-inflammatory cytokines, like IL-10 and TGFβ, and chemokines CCL17, CCL22, and CCL24. In terms of metabolic status, M1 macrophages produce inducible nitric oxide synthase (iNOS) enzyme to catalyze L-arginine to reactive nitrogen intermediates (RNI), which promote the killing of pathogens. On the contrary, M2 macrophages stimulate arginase I (Arg I) enzyme to catalyze L-arginine into ornithine and polyamines that support tissue remodeling and fibrosis (11).

Immune response modulation is a potential therapeutic intervention. Many synthetic RIG-I agonists that activate antiviral defense have been tested as probes, used to delineate mechanisms, and tested as pharmacological agents to combat IAV infection (12–14). A family of short synthetic, triphosphorylated stem-loop RNAs (SLRs) have been designed to activate the RIG-I pathway and induce a potent interferon (IFN) response (15). With a stable RNA tetraloop blocking the opposite end of the SLR duplex, RIG-I is guaranteed to bind the SLR triphosphorylated duplex terminus. They offer useful resources for examining the mechanism that causes RIG-I activation. Early studies are promising as one of the molecules, SLR14, demonstrated remarkable prophylactic protective capacity against lethal severe acute respiratory syndrome coronavirus 2 (SARS−CoV−2) infection in a mouse model (16).

In this report, we chose to study a similar molecule, SLR10, which has been demonstrated to be a highly potent activator of the IFN response in mice (15). We tracked the lung innate immune response to IAV infection in nonsmoking (NS) and CS-exposed mice after SLR10 administration. We addressed whether RIG-I pathway activation with SLR10 leads to protection of NS and CS-exposed mice after IAV infection.

## Materials and methods

### Ethics statement

The animal study was reviewed and approved by The Institutional Animal Care and Use Committee (IACUC) of the University of Oklahoma Health Sciences Center (protocol number: 17-106-HI). The facility where this research was conducted is accredited by AAALAC.

### Influenza A virus

The IAV strain used in this study was A/PR/34/8 (PR8). The stocks were propagated in Madin-Darby canine kidney (MDCK, ATCC, Manassas, VA) cells following standard procedures (17). The virus was titered by plaque assay in MDCK cells, aliquoted, and stored at −80°C.

### Whole-body CS exposure, SLR10 administration, and influenza virus infection

Unrestrained C57BL/6 mice were subjected to whole-body exposure of the smoke from 1R6F reference cigarettes (University of Kentucky, Lexington, KY) for 4 hours per day for 6 weeks, as previously described (4).

SLR10 was obtained from the Anna Pyle lab in Yale University. Mice under SLR10 administration and IAV infection were anaesthetized by isoflurane inhalation. The mice were treated with SLR10 (~1.1mg/kg) intratracheally in complex with *in vivo*-JetPEI (PolyPlus, France) in a 50-µl volume 1 day prior to infection with A/Puerto Rico/8/1934 (PR8) mouse-adapted IAV. The negative control group was injected with the same amount *in vivo*-JetPEI only or pppNS in *in vivo*-JetPEI. The mice were held in a vertical position while sedated and administered by intranasal instillation of virus diluted in PBS (50 µl solution). An equal volume of PBS without virus was sham inoculated to the mock group. The animals were meticulously watched both during and after each procedure to make sure they recovered properly. Mice were monitored daily for 15 days for clinical symptoms (shaking, inactivity, and piloerection) and their weight was recorded daily.

### Collection of primary human bronchial epithelial cells and human alveolar macrophages

According to a procedure authorized by the Institutional Review Board of the University of Oklahoma Health Sciences Center (IRB # 2197), human bronchial epithelial cells (HBEC) were collected by bronchoscopy and bronchial brushing from healthy, non-smoking adult volunteers, as previously described (18). Whole human donor lungs were obtained through the International Institute for the Advancement of Medicine (Edison, NJ), a nonprofit division of the Musculoskeletal Transplant Foundation, or from LifeShare, a nonprofit organ procurement organization in Oklahoma City, OK. Human alveolar macrophages were isolated from the lungs as previously described (19).

### SLR10 transfection into cells

SLR10 was diluted in 250 μl Opti-MEM Reduced Serum Medium without serum and combined with an equal volume of a 2% solution of Lipofectamine in Opti-MEM after a short incubation of both solutions at room temperature. Then, after an additional 20 minutes of incubation, SLR10-Lipofectamine 2000 complexes were added to wells containing cells and medium with SLR final concentration at 5 µg/ml. The cells were then incubated for 37°C in a CO_2_ incubator prior to harvest.

### Multiplex immunoassay

Using multiplex immunoassay, the cytokine protein levels in mouse bronchoalveolar lavage fluids (BALF) were assessed (Eve Technologies, Calgary, AB, Canada). For disinfection, each sample was diluted two-fold in 1% triton X-100 (final).

### Measurement of mRNA expression by quantitative real-time PCR

A modified TRIzol (Invitrogen, Carlsbad, CA) procedure was used to extract and quantify the total RNA from the lung. The electrophoresis of formaldehyde on agarose gel was used to confirm the integrity of the RNA. Using the oligo (dT) SuperScript II First-Strand Synthesis System for RT-PCR, equal amounts (1µg) of RNA from each sample were reverse-transcribed into cDNA (Invitrogen, Carlsbad, CA). Gene specific primers’ sequences are shown in **Table 1**. Additional primers’ sequences were those used in our prior publication (6). qRT-PCR was carried out on a Bio-Rad CFX96TM Touch Real-Time PCR Detection System using 100 ng sample RNA and SYBR Green (Quanta Biosciences, Gaithersburg, MD). The target gene’s ΔCT value and its normalizer, β-actin, were used to calculate and plot the results.

**Table 1:** List of primers used in RT-PCR.

Gene	Forward primer (5’-3’)	Reverse primer (5’-3’)
human iNOS	GTTCTCAAGGCACAGGTCTC	GCAGGTCACTTATGTCACTTATC
human CCL18	GGTGTCATCCTCCTAACCAAGAGA	GCTGATGTATTTCTGGACCCACTT
human CD206	GCAAAGTGGATTACGTGTCTTG	CTGTTATGTCGCTGGCAAATG
human CXCL9	GTGGTGTTCTTTTCCTCTTGGG	ACAGCGACCCTTTCTCACTAC
human Arg1	TGATGTTGACGGACTGGACC	ATCTAATCCTGAGAGTAGCCCTGT
mouse GM-CSF	ACCACCTATGCGGATTTCAT	TCATTACGCAGGCACAAAAG
mouse IL-1β	CCTTCCAGGATGAGGACATGA	TGAGTCACAGAGGATGGGCTC

### Histological analysis of mouse lung

Mice were killed five days after the IAV infection, and the lungs were fixed in 4% paraformaldehyde in PBS for 24 hours at room temperature before being embedded in paraffin. Hematoxylin and eosin (H & E) staining was performed on fixed tissue to evaluate fibrosis and inflammation. Lung tissues were histologically evaluated for alveolar damage (e.g. pneumocyte necrosis or hyaline membrane formation), serous exudate/edema, peribronchial inflammation, alveolar fibrin deposition, alveolar and interstitial inflammatory infiltrates, perivascular infiltrates, perivascular edema, and hemorrhage. All tissues were assigned a quantitative histopathological score based on previously documented criteria (20, 21): 0 = no apparent pathology/change; 1 = minimal change (minimally increased numbers of inflammatory cells); 2 = mild change (mild inflammatory infiltrates, alveolar damage/necrosis, fibrin deposition, and/or exudation); 3 = moderate change (as previously described, but moderately more extensive); and 4 = marked changes (as previously described, but with severe inflammation, alveolar damage, hyaline membrane formation, necrosis, exudation, or hemorrhage). To eliminate bias and ensure scientific rigor, all tissues were evaluated and scored by a board-certified veterinary pathologist who was blinded to the study groups.

### Statistical analysis

Statistical significance was determined by one-way ANOVA with Student-Newman-Keuls *post hoc* correction for multiple comparisons. The logrank test was used to determine the significance of the survival rate. The p value for RT-PCR results was calculated using the ΔCt values from different experimental groups. Significance was considered as p < 0.05.

## Results

### SLR10 administration induced innate immune responses in human primary bronchial epithelial cells and in mice

**Figure 1:**
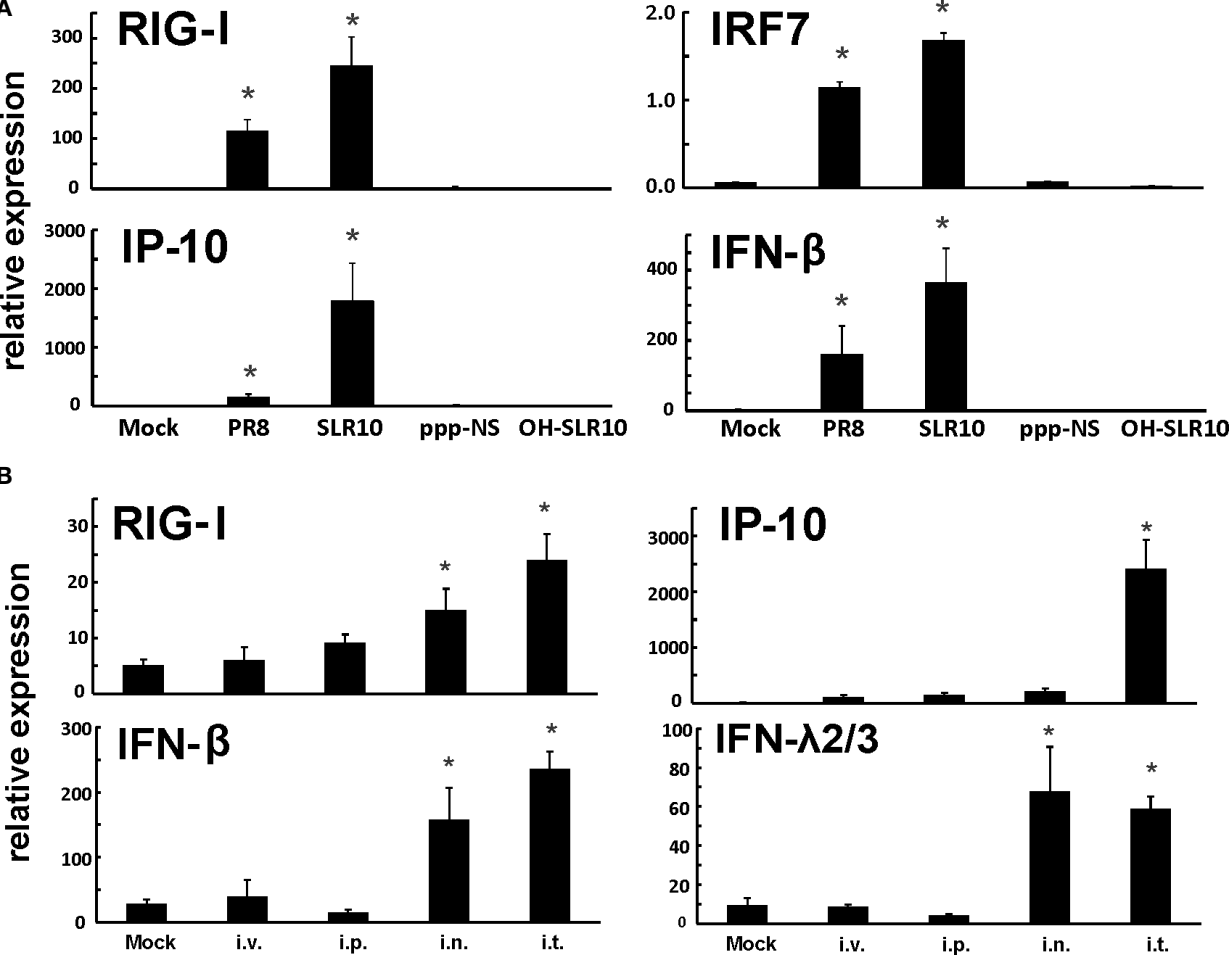
SLR10 administration induced robust RIG-I initiated innate immune responses in HBECs and mouse lung. **(A)** Cells were stimulated by 5 µg/ml of SLR10. IAV PR8 infection served as a positive control. OH-SLR10 and ppp-NS served as negative controls. Cells were collected at 6 h after SLR10 stimulation or 24 h after PR8 infection. **(B)** C57BL/6 mice were administrated with 1.1 mg/kg of SLR10 with *in vivo*-JET PEI mixture by four administration routes, intravenously (i.v.), intraperitoneally (i.p.), intranasally (i.n.), and intratracheally (i.t.). After 24 h, mouse lungs were collected. mRNA levels were assessed by qRT-PCR and normalized to β-actin. Statistical significance was determined by one-way ANOVA with Student-Newman-Keuls *post hoc* correction for multiple comparisons. *denotes significant difference between the noted group and mock group, p<0.05. Bar graph represents mean ± standard error (n=4).

First, we confirmed that SLR10 activates RIG-I and cytokine responses using HBECs. Cells were stimulated by SLR10 and IAV PR8 infection was served as a positive control. We also included two negative controls, OH-SLR10 (RNA stemloops lacking 5’-phosphatemoieties) and ppp-NS (a 5’-triphosphorylated single-stranded RNA that is the same length as SLR10 but nonstructural). Similar to PR8 infection, SLR10 significantly increased mRNA levels of RIG-I and the downstream transcription factor IRF7 in these cells. In addition, mRNA levels of RIG-I induced IFN-β and IP-10 were increased 400 and 2000-fold over mock, respectively (**Figure 1A**). In addition, SLR10 induced IP-10 was 11-fold higher than virus induced IP-10. By contract, there was no RIG-I and cytokine induction in OH-SLR10 and ppp-NS control-treated cells. Thus, SLR10 is a highly potent activator of RIG-I and IFN *in vitro* in human airway epithelium.

We used a proven RNA delivery technique to inject RNA ligands into living animals in order to test SLR10’s capacity to activate the RIG-I pathway in mouse lungs. We compared four administration routes, namely intravenously (i.v.), intraperitoneally (i.p.), intranasally (i.n.), and intratracheally (i.t.), in wild-type (WT) C57BL/6 mice. Lungs were collected 24 h after administration of a complex of RNA ligands and polyethylenimine (JetPEI). SLR10 administration induced innate immune responses in these animals (**Figure 1B**). We found that i.t. administration showed durable mRNA induction of RIG-I, IP-10, IFN-β, and IFN-λ2/3 in the lung at 24 h after SLR10 treatment. In contrast, i.p. administration demonstrated the lowest relative mRNA expression of antiviral genes of the routes tested. Therefore, i.t. administration is the most effective way to induce enduring innate immune responses in the mouse lung. We exclusively used this method in the following experiments.

**Figure 2:**
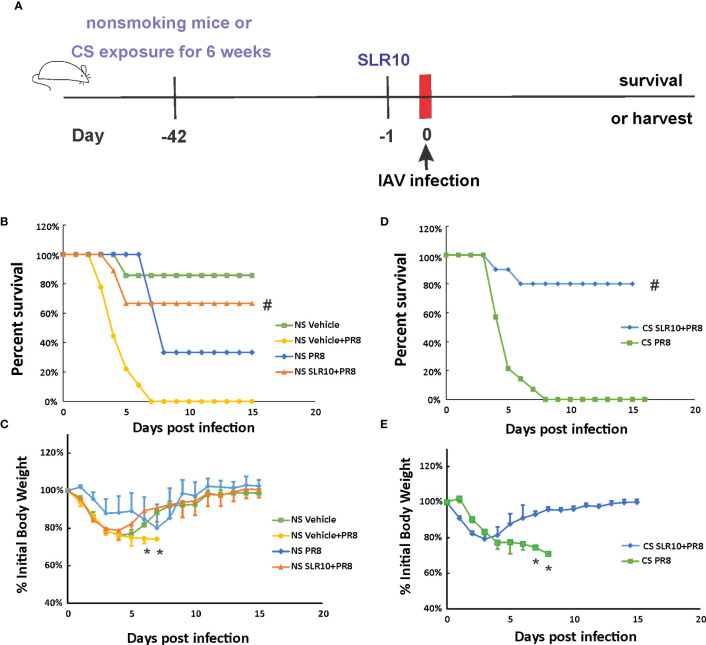
SLR10 administration increased survival rates of both NS and CS-exposed mice in lethal IAV infection. **(A)** Schematic of the experimental plan of SLR10 administration and IAV infection in NS and CS mice. CS mice were exposed to CS for 6 weeks. For SLR10 treatment groups, the mice were treated with 1.1 mg/kg of SLR10/*in vivo*-JET PEI intratracheally 1 day before IAV infection. The mice were intranasally inoculated with IAV at 1000 PFU/mouse. Mortality and body weight were monitored daily. Body weight data were normalized to each mouse’s starting body weight. Data are expressed as mean ± standard deviation [n≥9 for all groups except NS vehicle (n=7)]. Mortality **(B)** and body weight **(C)** during lethal IAV infection in SLR10 administrated NS mice. ^#^denotes significant survival rate difference between the NS SLR10+PR8 and NS vehicle+PR8 groups, p<0.05. *denotes significant weight loss difference between the NS SLR10+PR8 and NS vehicle+PR8 groups, p<0.05. Mortality **(D)** and body weight **(E)** during lethal IAV infection in SLR10 administrated CS mice. The logrank test was used to determine the significance of the survival rate. ^#^denotes significant survival rate difference between the CS SLR10+PR8 and CS PR8 groups, p<0.05. *denotes significant weight loss difference between the CS SLR10+PR8 and CS PR8 groups, p<0.05.

Mice were given 1.1 mg/kg of SLR10/JetPEI 1 day prior to IAV infection to test whether SLR10 administration improves survival in NS mice. (**Figure 2A**). The negative control group was injected with the same amount *in vivo*-JetPEI vehicle only. IAV-infected animals were inoculated with a lethal dose of virus (1000 PFU), which is a LD_50_ dose in NS mice (causing approximately 50% mortality). Survival and weight loss were monitored over the course of 15 days. When mice died in the cage or reached 70% of their original body weight, their deaths were recorded. JetPEI/PBS vehicle administration plus sham inoculation (NS vehicle group) in mice caused an adverse effect in these animals, evidenced by initial weight loss of all mice in this group and death of one animal. Despite that, SLR10 administration significantly decreased overall mortality in NS mice during IAV infection as assessed by logrank test (**Figure 2B**). Specifically, SLR10-treated mice had better survival (NS SLR10+PR8, 67%) compared to untreated animals (NS vehicle+PR8, 0%). The body weight data correlated with mortality data in that lower survival groups lost more weight than higher survival groups. Of note, the maximum body weight loss of SLR10 treated group infected with IAV (21% loss) was significantly less than in the IAV-infected untreated group (26% loss; **Figure 2C**). The survival rate of NS PR8 is 33% and NS vehicle+PR8 is 0%. So, NS vehicle+PR8 group had 33% less survival rate compared to NS PR8. The difference with or without vehicle is wider than NS vehicle only, which had a survival rate of 86%. Thus, we suspect that vehicle plus PR8 might have synergized negative effects on mouse survival rate. Despite the negative effect of vehicle+PR8, SLR10 (in vehicle)+PR8 mice still have a significantly better survival rate than vehicle+PR8 group. The result further demonstrated that SLR10 not only helped protect against IAV induced mortality but the effect was so beneficial that it negated or overcame the negative effects of the synergized negative effects of vehicle+PR8.

Following that, we examined whether SLR10 administration affected mortality and weight loss during IAV infection in CS-exposed mice. Whole-body CS exposure was performed as described (4). Unrestrained mice were subjected to 6 weeks of CS exposure for 4 hours per day in a whole-body cigarette smoking chamber system (Teague Enterprises, Davis, CA). Some of the mice were given SLR10 intratracheally after a 6-week CS exposure. The mice were then all given a lethal dose of the PR8 virus (**Figure 2D**). As previously demonstrated, CS exposure increased the morbidity and mortality of IAV infection in mice (6, 22). In **Figure 2D**, after IAV infection (1000 PFU/mouse), the infection caused death in all CS-exposed mice (0% survival for CS mice). However, SLR10 administration to CS-exposed mice improved survival rate during IAV infection (80% survival for CS SLR10+PR8 group vs.. 0% for CS PR8 group, p<0.05 by Logrank test). Morbidity was also decreased by SLR10 treatment (**Figure 2E**), as treated mice had less weight loss compared with diluent treated mice (21% loss for CS SLR10+PR8 group vs. 29% loss for CS PR8 group, p<0.05; **Figure 2E**). Thus, SLR10 significantly decreased weight loss and improved survival in both NS and CS-exposed mice during IAV infection.

### SLR10 restored inflammatory responses in CS-exposed mice but did not increase lung injury in NS mice during IAV infection

**Figure 3:**
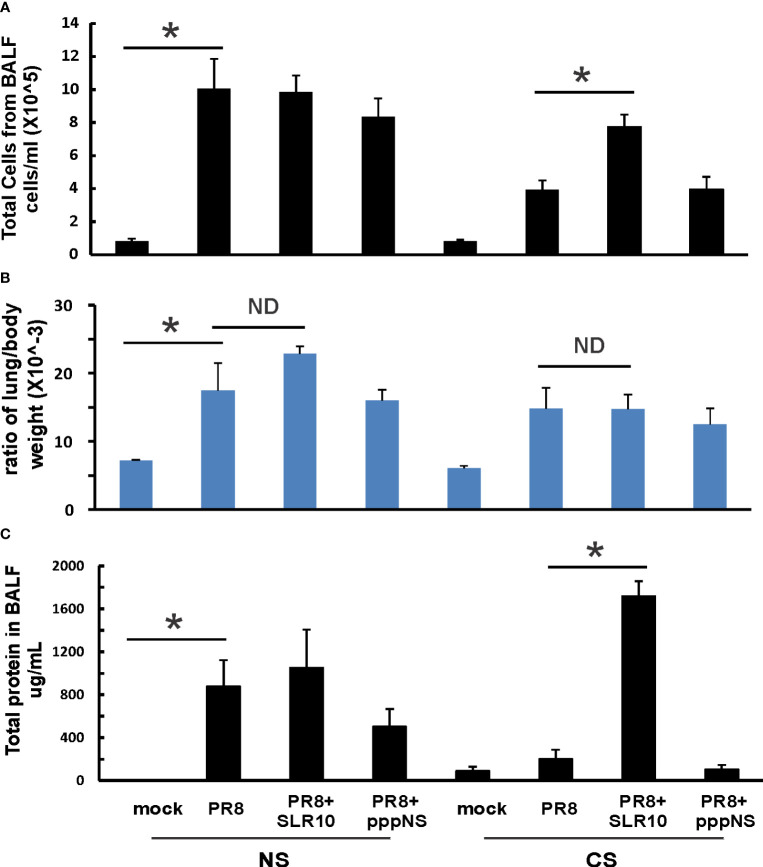
Lung injury and cellularity in the lungs. CS exposure, SLR10 administration, and IAV infection are the same as in **Figure 2A**. Each mouse was infected with 500 PFU of IAV. Mock-treated mice were inoculated with PBS. Bronchoalveolar lavage fluid (BALF) or lung tissue was harvested at day 5 after infection. Total immune cells **(A)**, total protein concentration **(C)** in BALF, and ratio of lung/body weight **(B)** were determined. Data are expressed as means ± SEM (n≥4/group). Statistical significance was determined by one-way ANOVA with Student-Newman-Keuls *post hoc* correction for multiple comparisons. ^*^denotes significant difference between the two groups, p<0.05. ND, no significant difference between the two groups.

Mice were intranasally inoculated with IAV at 500 PFU/mouse to test the effect of SLR10 on the inflammatory response to IAV. At 5 days p.i., animals were sacrificed by an isoflurane overdose. The total number of inflammatory cells in bronchoalveolar lavage fluids (BALF)was first determined. IAV infection increased the total viable leukocytes in BALF in mice, as expected (**Figure 3A**; NS mock vs.. NS PR8). As previously demonstrated (6), CS exposure reduced the total BALF cell number during IAV infection (CS PR8 vs. NS PR8). SLR10 administration to CS mice (CS PR8+SLR10), on the other hand, significantly increased total BALF cell numbers compared to CS PR8 mice (**Figure 3A**). These results showed that SLR10 increased immune cell influx into the lung in CS-exposed mice. These results showed that SLR10 increased immune cell influx into the lung in CS-exposed mice back to levels seen in NS mice during IAV infection (NS PR8 vs. CS PR8+SLR10). The restored inflammation in the lung was also confirmed by the total amount of protein in the BALF, which also showed that the CS PR8+SLR10 group had much more protein than the CS PR8 group (**Figure 3C**).

The lung-to-body weight ratio is an important predictor of lung injury. Mice infected with IAV had a significantly higher lung-to-body weight ratio (**Figure 3B**; NS vs. Mock). SLR10 treatment to NS mice did not significantly change the ratio over that seen in the untreated group (NS PR8+SLR10 vs. NS PR8). Meanwhile, SLR10 administration to CS-exposed mice did not cause more lung injury than in untreated CS mice during IAV infection (**Figure 3B**; CS PR8+SLR10 vs. CS PR8). Thus, SLR10 specifically restored inflammatory cell recruitment in the lungs of CS-exposed IAV infected mice (**Figure 3A**) but did not lead to enhanced lung injury compared to untreated NS and CS mice (**Figure 3B**) during IAV infection. This occurred despite more protein in BALF in the SLR10-treated IAV infected CS mice than in untreated IAV-infected CS mice (**Figure 3C**).

### Effect of SLR10 on lung histopathology during IAV infection

**Figure 4:**
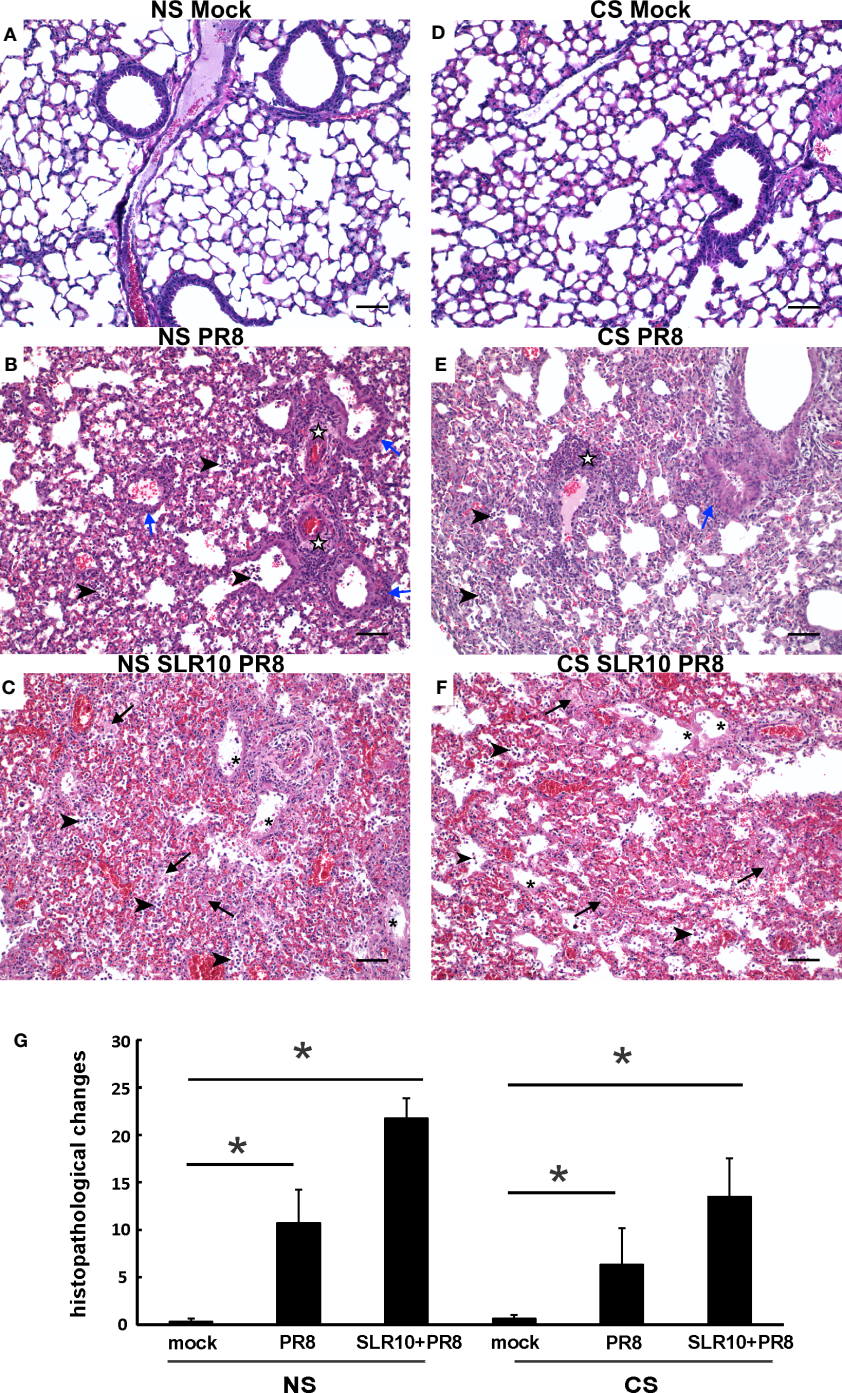
Effect of SLR10 on pulmonary histopathology during IAV infection in NS and CS-exposed mice. CS exposure, SLR10 administration, and IAV infection are the same as in **Figure 2A**. Each mouse was infected with 500 PFU of IAV. Mock-treated mice were inoculated with PBS. Animals were sacrificed at 5 days after infection and lung tissue was harvested. Lung tissue sections prepared from the infected mice were fixed, processed, and stained with hematoxylin–eosin **(A-F)**. Histopathologic evaluation and scoring of IAV infection were determined by a blinded pathologist **(G)**. Compared to the open alveolar spaces in healthy lungs of uninfected of mice with no CS **(A)**, NS Mock), the lungs of mice with IAV infection **(B)**, NS PR8) contained bronchiolar inflammatory infiltrates (blue arrows) that frequently spilled over into the adjacent alveolar spaces (arrowheads). The interstitial space surrounding small and large caliber vessels was also expanded by perivascular edema and lymphocytic infiltrates (white stars). Lesions in the lungs of IAV-infected mice treated with SLR10 **(C)**, NS SLR10 PR8) were more severe and featured distinct evidence of diffuse alveolar damage (DAD) characterized by denuded bronchial epithelium (asterisks), alveolar inflammatory infiltrates (arrowheads), and marked intra-alveolar fibrin (arrows). The lungs of healthy, uninfected mice subjected to CS **(D)**, CS Mock) were also histologically normal, with open bronchi/alveoli and minimal alveolar edema. Pulmonary lesions in IAV-infected mice **(E)**, CS PR8) subjected to CS were less severe than in mice without smoke **(B)**, NS IAV), although features such as alveolar inflammation (arrowheads), peribronchiolar infiltrates (blue arrows), and perivascular inflammation and edema (white stars) were readily observed. Similar to **(C)** the lungs of IAV-infected mice treated with SLR10 and subjected to CS **(F)**, CS SLR10 PR8) exhibited widespread alveolar damage with hyaline membrane formation (asterisks), as well as alveolar inflammatory infiltrates (arrowheads), and marked intra-alveolar fibrin (arrows). Scale bar = 50 µM (20X). Statistical significance was determined by one-way ANOVA with Student-Newman-Keuls *post hoc* correction for multiple comparisons. *denotes significant difference between the two groups, p<0.05. The image shown is representative of five mice lungs from each group.

Lung tissues were evaluated for histopathological changes. As shown in **Figure 4**, the lungs of healthy, mock-infected mice with CS (CS Mock) and without CS (NS Mock) were histologically normal. Both groups of IAV-infected mice (NS PR8 and CS PR8) exhibited similar histopathologic lesions characterized predominately by multifocal areas of bronchiolar inflammation accompanied by accumulations of inflammatory infiltrates within adjacent alveolar spaces. Moderate edema and moderate-to-large numbers of small lymphocytes and plasma cells frequently expanded the perivascular space surrounding numerous small and large caliber vessels. Treatment with SLR10 seemed to increase the severity of pathologic lesions in both CS (CS SLR10 PR8) and NS (NS SLR10 PR8) mice, though it did not reach statistical significance, and it may reflect enhanced inflammatory recruitment. SLR10-treated and untreated mice in both CS exposure groups exhibited evidence of diffuse alveolar damage (DAD), characterized by bronchial epithelium necrosis and ulceration with occasional hyaline membrane formation. The alveoli in all infected mice were frequently filled with large amounts of fibrin admixed with inflammatory cells (neutrophils and macrophages), hemorrhage, and edema. The interstitial space surrounding pulmonary vessels was frequently expanded by perivascular edema and lymphocytic infiltrates. No significant differences in histologic score were observed for any group based on the presence/absence of CS (for example NS PR8 vs. CS PR8). Thus, SLR10 appeared to result in higher overall inflammatory scores than untreated groups in both NS and CS mice at 5 days following infection (**Figure 4G**).

### SLR10 administration to mice promoted M1 macrophage polarization in the lung during influenza infection

**Figure 5:**
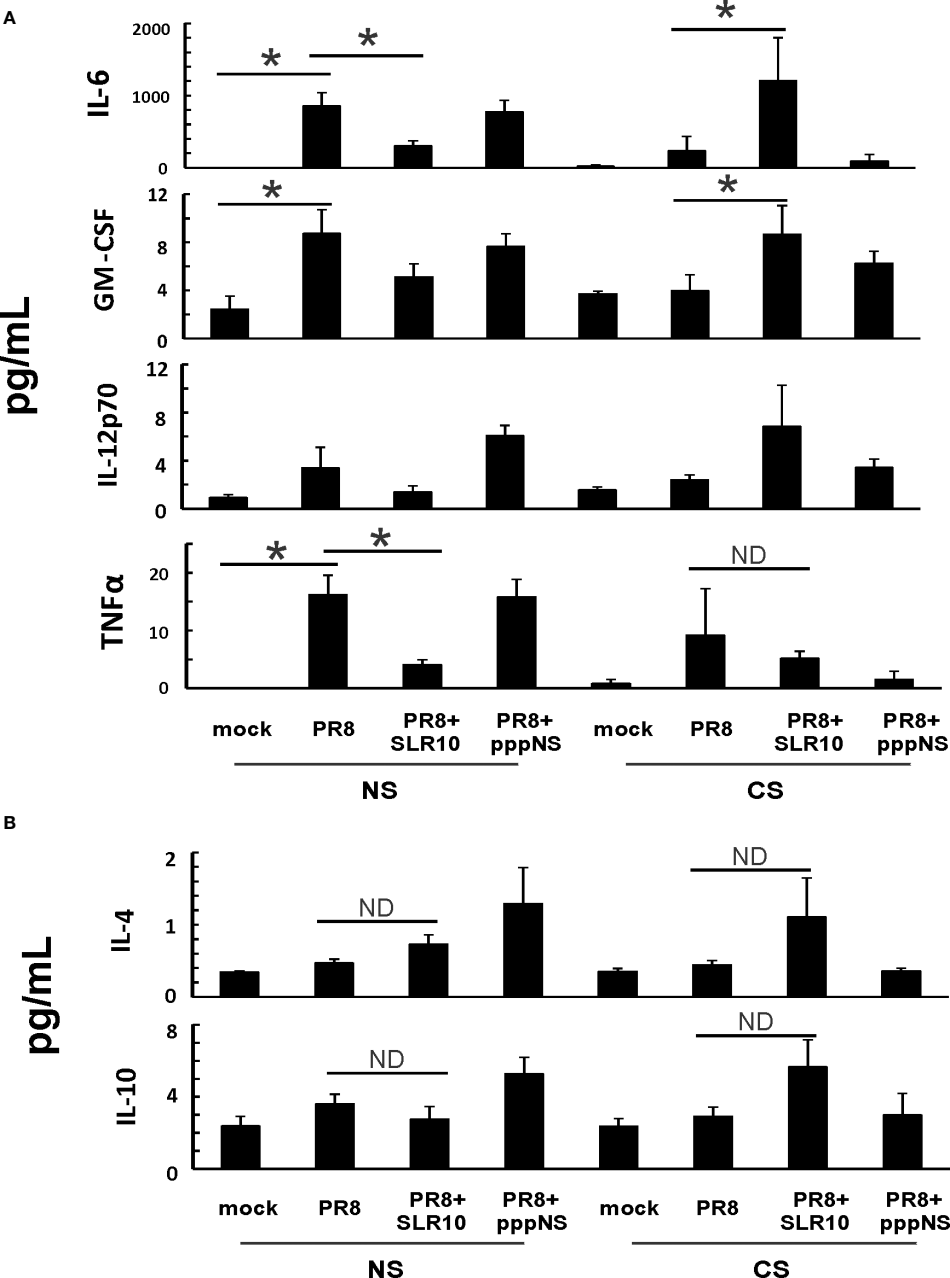
SLR10 administration to IAV-infected CS-exposed mice increased proinflammatory cytokine levels in BALF. CS exposure, SLR10 administration, and IAV infection are the same as in **Figure 2A**. Mice were infected with 500 PFU of IAV. BALF were harvested at day 5 post infection. Mock-treated mice were inoculated with PBS. Cytokine protein levels were determined by multiplex immunoassay. Data are expressed as mean ± SEM (n ≥ 4 per group). Statistical significance was determined by one-way ANOVA with Student-Newman-Keuls *post hoc* correction for multiple comparisons. * denotes significant difference between the two groups, p<0.05. ND, no significant difference between the two groups.

To characterize cytokine induction during infection in all groups, we examined cytokine protein levels in BALF using multiplex immunoassay (**Figure 5**). For IFNs, CS exposure suppressed IFN-α, IFN-β, and IFN-γ induction by IAV relative to that observed in NS mice (CS PR8 *vs*. NS PR8, **Supplemental Figure 1**). Interestingly, we did not see enhanced IFN induction in SLR10-treated mice at this time point post-infection (p.i.). We also examined effects of CS and SLR10 on proinflammatory cytokine induction by IAV. IL-6 and GM-CSF induction by virus was significantly suppressed by CS exposure, as we have previously demonstrated. Remarkably, proinflammatory cytokines IL-6 and GM-CSF in response to IAV were enhanced in the CS SLR10 group (**Figure 5A**). IL-12 levels also appeared to be increased in CS SLR10 mice although this did not reach statistical significance. In the case of anti-inflammatory cytokines, IAV infection did not increase IL-4 or IL-10 protein levels in either NS or CS mice (**Figure 5B**). Their levels seemed to be enhanced in CS SLR10 mice infected with IAV, but this did not reach statistical significance. Thus, in CS-exposed mice, SLR10 clearly increased proinflammatory cytokine production and did not significantly increase anti-inflammatory cytokine production in these animals.

**Figure 6:**
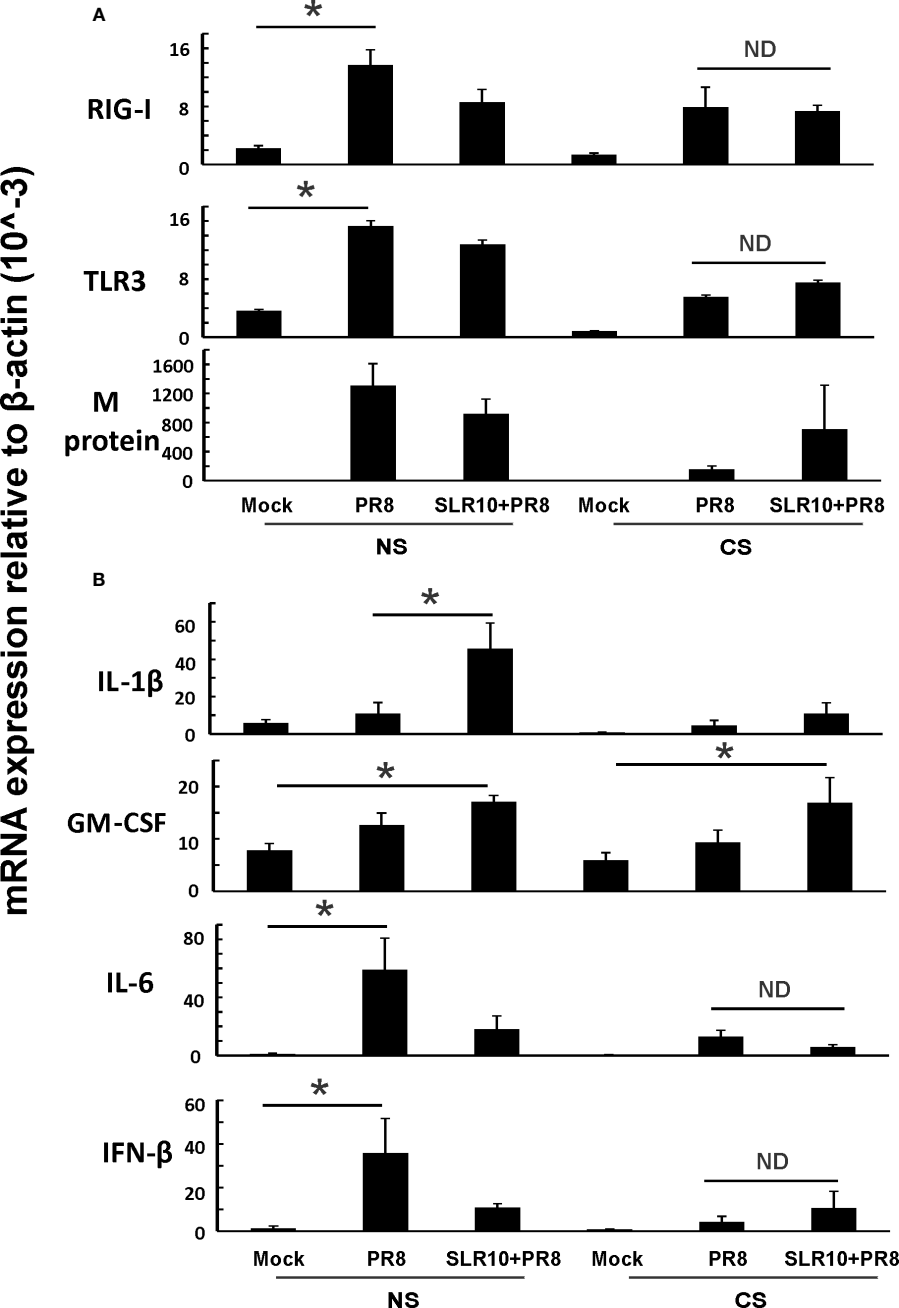
Innate immune responses to influenza infection in mouse lung. CS exposure, SLR10 administration, and IAV infection are the same as in **Figure 2A**. Mice were infected with 500 PFU of IAV. The mice were sacrificed at day 5 p.i. and **(A)** RIG-I, TLR3, and IAV M protein gene **(B)** cytokine IL-6, IFN-β, IL-1β, and GM-CSF mRNA levels in the lungs were assessed by qRT-PCR and normalized by β-actin levels. Data are expressed as mean ± SEM (n =5). Statistical significance was determined by one-way ANOVA with Student-Newman-Keuls *post hoc* correction for multiple comparisons. * denotes significant difference between the two groups, p<0.05. ND, no significant difference between the two groups.

Next, we examined lung tissues in mice 5 days p.i. and determined mRNA levels of PRRs and corresponding downstream cytokines by qRT-PCR. CS-exposed mice had suppressed RIG-I and TLR3 induction by IAV compared NS mice (**Figure 6A**; CS PR8 vs. NS PR8), as we have previously demonstrated (6, 22). SLR10 administration did not change RIG-I and TLR3 mRNA induction by virus infection in the lungs of CS mice (**Figure 6A**; CS SLR10 PR8 vs. CS PR8). In terms of viral RNA expression, all IAV-infected groups had similar IAV M protein mRNA expression showing similar viral tissue burden; however, the CS PR8 group, surprisingly, had lower M protein replication than other infected groups. This is consistent with our previous publication (6) that there was no direct correlation of mortality with lung viral burden (**Figure 6A** last panel). GM-CSF mRNA levels were higher in SLR10-treated groups regardless of CS exposure (**Figure 6B**). However, we did not find that IL-6 mRNA in CS SLR10 PR8 was higher than the CS PR8 group. Therefore, we did not find significant changes related to inflammation except for GM-CSF in whole lung tissue which mainly represents epithelial cell gene expression.

**Figure 7:**
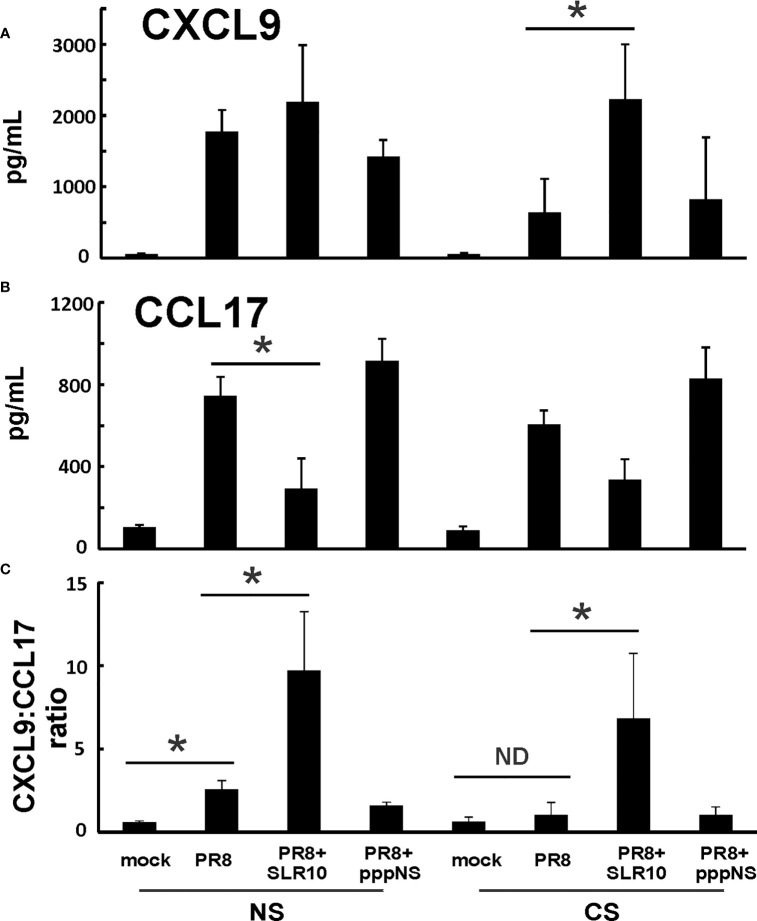
CXCL9:CCL17 ratio in BALF indicated an M1 macrophage polarization state directed by SLR10. CS exposure, SLR10 administration, and IAV infection are the same as in **Figure 2A**. Mice were infected with 500 PFU of IAV. BALF were harvested at day 5 p.i. Mock-treated mice were inoculated with PBS. Cytokine CXCL9 **(A)** and CCL17 **(B)** protein levels were determined by multiplex immunoassay and CXCL9:CCL17 ratios in BALF were calculated **(C)**. Data are expressed as mean ± SEM (n ≥ 4 per group). Statistical significance was determined by one-way ANOVA with Student-Newman-Keuls *post hoc* correction for multiple comparisons. *denotes significant difference between the two groups, p<0.05. ND, no significant difference.

Since GM-CSF, IL-6, and IL-12 cytokines are associated with M1 macrophages, we suspected that altered alveolar macrophage polarization may be responsible for the changes seen both in CS exposure and in SLR10-treated groups. We next measured chemokines CXCL9 and CCL17 protein levels in BALF by ELISA. These two cytokines are important markers for M1 and M2 macrophage polarization, respectively. CXCL9 was significantly induced by SLR10 even in CS-exposed mice (CS PR8+SLR10; **Figure 7A**). Meanwhile, CCL17 levels were lower in SLR10-treated groups compared to corresponding NS PR8 and CS PR8 groups (**Figure 7B**). We also examined the ratio of chemokine CXCL9 vs. chemokine CCL17, which is an intrinsic property that reveals M1 vs. M2 macrophage polarization (23). As expected, PR8 infection significantly increased the M1:M2 ratio in NS mice, indicating an M1 dominant profile during IAV infection (**Figure 7C**). CS exposure caused a shift towards M2 status in these animals (CS PR8 vs. NS PR8). Notably, SLR10 significantly increased the M1:M2 ratio in IAV-infected NS and CS mice (**Figure 7C**). Therefore, these results demonstrated CS favored M2 polarization during IAV infection while SLR10 administration to mice promoted alveolar macrophage polarization to an M1 phenotype.

### SLR10 stimulated human alveolar macrophage polarization to M1 phenotype *in vitro*

**Figure 8:**
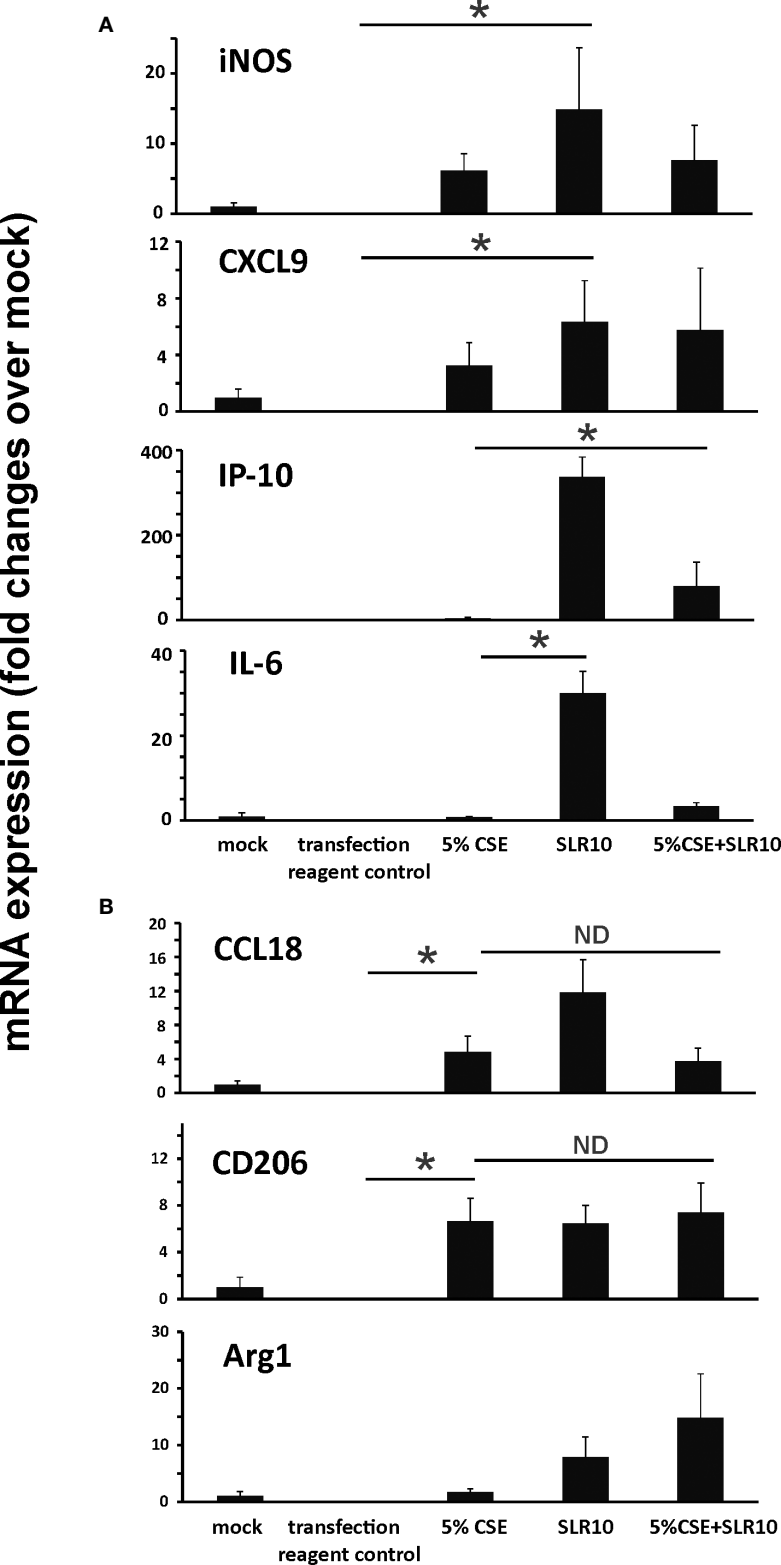
SLR10 induced human alveolar macrophages to an M1 polarization state even in CSE-treated cells. human alveolar macrophages were stimulated by 5 µg/ml of SLR10/Lipofectamine 2000. Transfection reagent only was used as a negative control. Cells were collected at 24 h after SLR10 stimulation. For CSE treatment, cells were incubated in media containing 5% CSE for 24 h. mRNA levels of **(A)** M1 macrophage markers and **(B)** M2 macrophage markers were assessed by qRT-PCR and normalized to β-actin. The data were presented as fold changes over mock. Bar graphs represents mean ± standard error (n=3). Statistical significance was determined by one-way ANOVA with Student-Newman-Keuls *post hoc* correction for multiple comparisons. *denotes significant difference between the two groups, p<0.05. ND, no significant difference between the two groups.

We have shown earlier that alveolar macrophages accounted for 95% of total BAL cells in uninfected mice and 55% in IAV-infected mice (6). We examined if SLR10 stimulation directed human alveolar macrophages to M1 macrophage polarization *in vitro*. The procedure should mimic what happens in the alveolar space in mouse lung after SLR10 administration intratracheally. Human alveolar macrophages were stimulated by SLR10 for 24 h. SLR10 significantly increased mRNA expression of M1 macrophage related proteins, such as iNOS, CXCL9, IP-10, and IL-6 (**Figure 8A**). In contrast, cigarette smoke extract (CSE) treatment induced expression of the M2 markers CCL18 and CD206 (**Figure 8B**), and to a much lesser extent, induced the M1 markers iNOS and CXCL9 (**Figure 8A**). However, the magnitude of mRNA induction by CSE for M2 markers was generally 10-100 fold higher than for the M1 markers. So, although CSE induced both M1 and M2 marker expression, the overall effect was to significantly drive macrophage polarization toward the M2 state. In a similar but opposite manner, though SLR10 did not reduce the mRNA expression of M2 markers, it stimulated expression of the M1 markers IP-10 and IL-6, resulting in a shift toward M1 polarization, even in CSE-treated macrophages. Thus, SLR10 not only stimulated human alveolar macrophage to M1 polarization, but it also reversed M2 polarization induced by CSE to M1 macrophage.

## Discussion

In this study, using a mouse model and a primary human cell culture model, we showed that SLR10 induced innate immune responses *in vivo and in vitro*. SLR10 significantly improved mortality in smoking mice during IAV infection by restoring the impaired proinflammatory response without causing additional lung injury. While CS exposure drove macrophages towards an M2 phenotype, SLR10 elevated the ratio of CXCL9: CCL17 in BALF, suggesting it polarized macrophages to classically activated M1 phenotypes. Using a human alveolar macrophage model, we found that SLR10 not only directed human alveolar macrophages to M1 phenotypes, but it also reversed CSE-induced M2 to M1 macrophage polarization. Our result was based on both mRNA levels (**Figures 6**, **8**) and protein levels (**Figures 5**, **7**). We used multiplex immunoassay and ELISA to detect protein levels in BALF, which is a direct way to monitor cytokine and chemokine changes in the lung.

SLR molecules are a set of stem-loop RNA that present a single duplex terminus and therefore bind to the RIG-I molecule, which mimic virus detection and activates the RIG-I pathway. Our result is consistent with an earlier report that showed RIG-I like receptors (RLRs) drive a signature of macrophage M1 polarization during West Nile virus (WNV) infection (24). In that report, the authors showed that the RLRs promote differential immune gene activation and response polarization to promote an M1/inflammatory signature while suppressing the M2/wound healing phenotype, using genome-wide RNA-seq and bioinformatics investigations of macrophages from mice. RIG-I activation during acute WNV infection leads peripheral monocytes and tissue-resident macrophages into an M1 phenotype through parallel regulation of ATF4/SMAD4 with canonical STAT signaling to mediate M1 gene induction and M2 gene suppression. Also, it has been reported that RIG-I signaling pathway activation by myoglobin promotes macrophage polarization to M1 type (25). Another whole-transcriptome analysis of human macrophages revealed that genes in significantly enriched pathways in response to IAV infection were specifically correlated with M1 macrophage subtype, and markedly up-regulated expression of RLR signaling pathway genes (26). It has been shown that CS promotes M2 polarization of macrophages (27) and nicotine causes immunosuppression *via* directing M2 polarization of macrophages (28). M2 macrophages are more vulnerable to IAV infection (29), have impaired phagocytic capacity (30), and induce less CD8+ T-cell response (31, 32). In our mouse model, CS polarized macrophages to an M2-like state, which is vulnerable to IAV infection. In our CSE-treated primary human alveolar macrophage model, CSE stimulated both M1 and M2 marker expression in these cells with disproportionate elevation of M2 markers. SLR10 stimulation increased M1 inflammatory responses and did not change M2; therefore, it reversed the macrophages to an M1 dominant phenotype, which are more protective in CS-exposed mice during IAV infection. Although SLR10 appeared to result in higher histopathological scores than untreated groups due to M1 polarization at 5 days following infection, mortality in the SLR10-treated groups was decreased. This is consistent with a prior study showing that intranasal pretreatment with Poly(I:C), a RIG-I and TLR3 agonist, accelerates inflammatory cell infiltration into lungs and protects aged mice from lethal IAV infections (33). Poly(I:C)-induced RIG-I pathway activation also inhibited vascular endothelial growth factor (VEGF) and provoked type 2 inflammatory tissue remodeling and ameliorated adaptive T-helper 2 (Th2)-mediated inflammation (34). Although it has been shown that Poly(I:C) converted tumor-associated macrophages to M1 polarization in cancer studies (35, 36), our current report is the first to demonstrate that RIG-I pathway activation by agonists directs alveolar macrophages to M1 phenotypes and augments type 1 inflammatory responses during viral infection. It is also the first to demonstrate that RIG-I pathway activation reverses deleterious M2 polarization induced by CS prior to viral infection and improves outcomes. While we propose that M1 polarization in the presence of CSE is the primary beneficial effect of SLR-10 based on both *in vivo* mouse and *in vitro* human data, it is also possible that suppression of the M2 phenotype, while maintaining the M1 phenotype, is the mechanism that leads to a more favorable M1:M2 profile early during influenza infection.

GM-CSF is a myeloid growth factor that promotes macrophage and dendritic cell proliferation and differentiation. *In vitro*, GM-CSF-treated macrophages have an “M1-like” phenotype, which have increased expression of proinflammatory cytokines (37). Furthermore, GM-CSF is thought to drive M1 polarization and to improve host defense functions and alveolar macrophage survival *in vitro* and in animal models *in vivo* (38, 39). In humans, inhaled GM-CSF promoted M1 polarization of alveolar macrophages and enhanced host defense without increasing neutrophil influx into the alveolar compartment (40). It has previously been shown that modulation of macrophage polarization by GM-CSF inhibited influenza infection (41). Our results showed that SLR10 administration increased GM-CSF mRNA and protein levels in the lung in both NS and CS mice. GM-CSF may play an important role in SLR10 mediated protection during lethal IAV infection.

The mechanisms underlying the severity of IAV infection in smokers are still being debated, with conflicting reports of both augmented inflammatory responses (42–44) and decreased antiviral host-defense (5, 45). During influenza infection, inflammation can either have supportive antiviral effects or can contribute to immunopathology (46). In order to maximize viral clearance while causing minimal damage to host cells, proper induction of innate responses is critical. We have demonstrated earlier that innate immune responses are suppressed by CS exposure (3, 4). SLR10 administration to CS-exposed mice helps restore the CS-suppressed proinflammatory responses but does not cause deleterious excessive inflammation and injury in the lung. Combined with our recent report that early IFN-β administration to CS-exposed mice improved outcomes during IAV infection (22), the proper proinflammatory cytokine responses in the host evidently would decrease the mortality in smokers during acute viral infection, especially at the early stage of infection.

Taken together, we conclude that SLR10 stimulation restored alveolar macrophage functions to their normal stressed responses, which increases inflammatory responses to IAV. This is especially critical for the CS-exposed host as this tends to shift macrophages into an M2-like phenotype. Thus, modulation of macrophage polarization by SLR10 might be a possible therapeutic strategy against IAV infection in smokers.

## Data availability statement

The original contributions presented in the study are included in the article/**Supplementary Material**. Further inquiries can be directed to the corresponding authors.

## Ethics statement

The studies involving human participants were reviewed and approved by the Institutional Review Board of the University of Oklahoma Health Sciences Center (IRB # 2197). The patients/participants provided their written informed consent to participate in this study. The animal study was reviewed and approved by The Institutional Animal Care and Use Committee (IACUC) of the University of Oklahoma Health Sciences Center (protocol number: 17-106-HI).

## Author contributions

WW and JM generated the hypothesis, performed statistical analysis, and wrote the manuscript. WZ, JB, JA, CM, and CX acquired the data. WW, CM, and JM contributed to writing the manuscript. All authors contributed to the article and approved the submitted version.

## Supplementary material

Supplementary Figure 1 Interferon levels in BALF in IAV-infected mice. CS exposure, SLR10 administration and IAV infection are the same as in **Figure 2A**. Mice were infected with 500 PFU of IAV. BALF were harvested at day 5 post infection. Mock treated mice were inoculated with PBS. Interferon protein levels were determined by multiplex immunoassay. Data are expressed as mean ± SEM (n ≥ 4 per group). * denotes significant difference between the two groups, p<0.05. ND = no significant difference between the two groups.
