# Supplementary figures and images for: RIG-I agonist SLR10 promotes macrophage M1 polarization during influenza virus infection
Source: Front Immunol. 2023 Jul 5;14:1177624. doi: 10.3389/fimmu.2023.1177624 (PMC10354434; doi:10.3389/fimmu.2023.1177624)

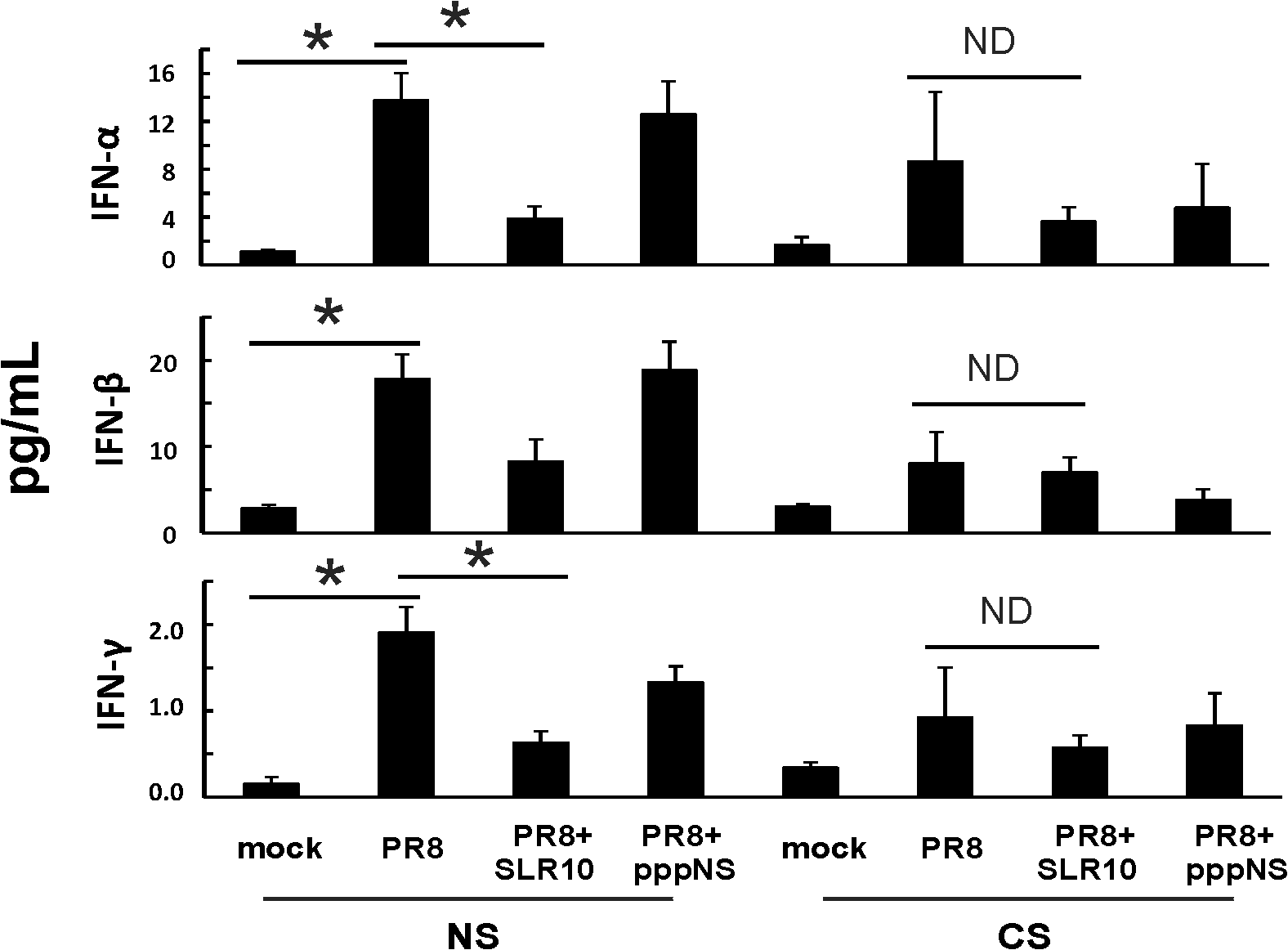

Supplement: Supplementary Figure 1 — Interferon levels in BALF in IAV-infected mice. CS exposure, SLR10 administration and IAV infection are the same as in Figure 2A. Mice were infected with 500 PFU of IAV. BALF were harvested at day 5 post infection. Mock treated mice were inoculated with PBS. Interferon protein levels were determined by multiplex immunoassay. Data are expressed as mean ± SEM (n ≥ 4 per group). * denotes significant difference between the two groups, p<0.05. ND = no significant difference between the two groups. [file Image_1.tif]
